# The ischemia‐enhanced myocardial infarction protection‐related lncRNA protects against acute myocardial infarction

**DOI:** 10.1002/mco2.632

**Published:** 2024-07-10

**Authors:** Rongzhou Wu, Tingting Wu, Qiaoyu Wang, Youyang Shi, Qianqian Dong, Xing Rong, Meiting Chen, Zhiyu He, Yu Fu, Lei Liu, Shuai Shao, Xueqiang Guan, Chunxiang Zhang

**Affiliations:** ^1^ Children's Heart Center The Second Affiliated Hospital and Yuying Children's Hospital of Wenzhou Medical University Institute of Cardiovascular Development and Translational Medicine The Second School of Medicine Wenzhou Medical University Wenzhou China; ^2^ Department of Biomedical Engineering The University of Alabama at Birmingham Birmingham Alabama USA; ^3^ Department of Cardiology Key Laboratory of Medical Electrophysiology Ministry of Education Institute of Cardiovascular Research Institute of Metabolic Diseases the Affiliated Hospital of Southwest Medical University Southwest Medical University Luzhou China

**Keywords:** acute myocardial infarction, apoptosis, cardiomyocyte, long noncoding RNA, MIPRL

## Abstract

Long non‐coding RNA RP11‐64B16.4 (**m**yocardial **i**nfarction **p**rotection‐**r**elated **l**ncRNA [MIPRL]) is among the most abundant and the most upregulated lncRNAs in ischemic human hearts. However, its role in ischemic heart disease is unknown. We found MIPRL was conserved between human and mouse and its expression was increased in mouse hearts after acute myocardial infarction (AMI) and in cultured human and mouse cardiomyocytes after hypoxia. The infarcted size, cardiac cell apoptosis, cardiac dysfunction, and cardiac fibrosis were aggravated in MIPRL knockout mice after AMI. The above adverse results could be reversed by re‐expression of MIPRL via adenovirus expressing MIPRL. Both in vitro and in vivo, we identified that heat shock protein beta‐8 (HSPB8) was a target gene of MIPRL, which was involved in MIPRL‐mediated anti‐apoptotic effects on cardiomyocytes. We further discovered that MIPRL could combine with the messenger RNA (mRNA) of HSPB8 and increase its expression in cardiomyocytes by enhancing the stability of HSPB8 mRNA. In summary, we have found for the first time that the ischemia‐enhanced lncRNA MIPRL protects against AMI via its target gene HSPB8. MIPRL might be a novel promising therapeutic target for ischemic heart diseases such as AMI.

## Graphical abstract

**M**yocardial **i**nfarction **p**rotection‐**r**elated **l**ncRNA (MIPRL) expression is increased in hearts after acute myocardial infarction (AMI) and in cultured cardiomyocytes after hypoxia. The upregulated MIPRL increases the expression of heat shock protein beta‐8 (HSPB8) by enhancing the stability of HSPB8 messenger RNA. MIPRL protects against AMI via HSPB8‐mediated inhibition of cardiac cell apoptosis and necrosis during AMI.

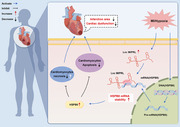

## 1 INTRODUCTION

Acute myocardial infarction (AMI) is the heart damage induced by acute and continuous ischemia and hypoxia. Despite modern therapeutics, AMI is still the main cause of death and disability worldwide due to our limited knowledge about the key pro‐ and anti‐damage molecules under AMI conditions.^1^, ^2^, ^3^, ^4^ Thus, uncovering the molecular mechanisms in the pathogenesis of AMI and identifying new molecular therapies for the serious disease are one of the hot research fields in cardiology.^1^, ^2^, ^3^, ^4^

In the past two decades, major progress in medical research has been the discovery of non‐coding RNAs (ncRNAs), which is a new supplement to the central dogma (DNA‐RNA‐Protein) of molecular biology. Although the ncRNAs cannot encode proteins directly, they do have strong biological functions by regulating protein expression via their target genes. Based on the research results in both pre‐clinical and clinical studies in the past 20 years, ncRNAs may play important roles in the development of many human diseases.^5^, ^6^ Targeting of ncRNAs is thus believed to be a promising novel therapeutic approach to many human diseases including cardiovascular diseases.^5^, ^6^

Among the ncRNAs studied, the long non‐coding RNAs (lncRNAs) are defined as ncRNAs longer than 200 nucleotides.^6^ In contrast to other non‐coding RNAs such as microRNAs, studies of lncRNAs in biomedicine are still in their early stages due to much more complex and diverse gene regulation mechanisms.^5^, ^6^ Another important feature of lncRNAs different from other ncRNAs is that the conservation of lncRNAs among species is very poor. It is thus a prerequisite to study a conserved lncRNA between humans and animals, especially for the pathogenesis and therapeutic studies of human diseases using animal models.

Based on the poor conservation of lncRNAs among species, one of the best research strategies for a lncRNA study could be that we first identify a conserved lncRNA in human disease tissues, and then use an animal model and cell model to test its roles in disease by using both the gain‐of‐function and loss‐of‐function approaches. To date, there are two studies performed on lncRNA profiling in human hearts with and without ischemia in vivo.^7^, ^8^ Among the lncRNAs that were differentially expressed between pairs of human heart samples before and after the ischemic insult, lncRNA RP11‐64B16.4 was found to be among the top three most abundant lncRNAs and was among the top six most up‐regulated lncRNAs in ischemic human hearts. More importantly, we used Clustal Omega,^9^ a multiple sequence program to generate alignments between human‐derived and mouse‐derived **m**yocardial **i**nfarction **p**rotection‐**r**elated **l**ncRNA (MIPRL) (sequencing analysis by RACE shown in Figure S1A). lncRNA MIPRL is conserved between humans and mice as shown by multiple conserved residues between the two sequences (Figure S1E). However, its biological function and the role of MIPRL in AMI have never been studied thus far. The aim of this study is to determine the role of this novel lncRNA in AMI and its potential mechanism. Based on the results of this study, we named it MIPRL.

## 2 RESULTS

### 2.1 The expression of MIPRL is increased in cardiomyocytes after hypoxia injury and in mouse hearts after AMI

**FIGURE 1:**
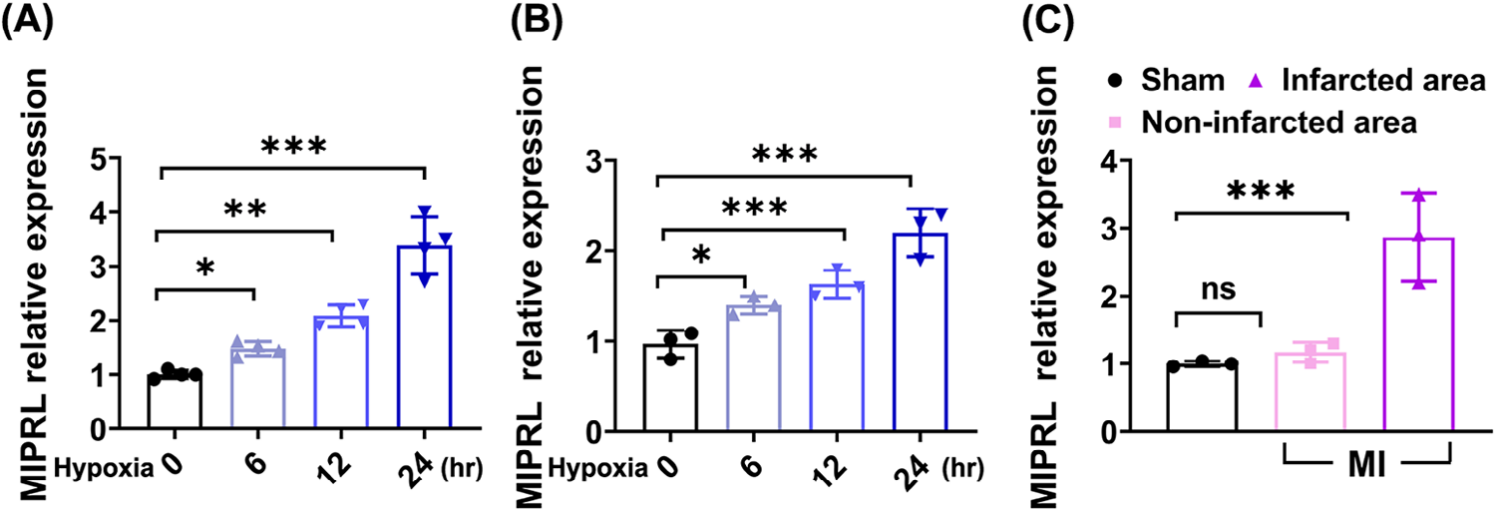
The expression of **m**yocardial **i**nfarction **p**rotection‐**r**elated **l**ncRNA (MIPRL) was increased in cardiomyocytes after hypoxia injury and in mouse hearts after AMI. (A)The human cardiomyocytes (HCM) were given hypoxia injury with 1% O_2_ and 5% CO_2_ at 37°C. Then cells were collected at different times and MIPRL expression was analyzed by quantitative real‐time polymerase chain reaction (qRT‐PCR) (*n* = 4). (B)The mouse cardiomyocytes isolated from neonatal mice were given hypoxia injury with 1% O_2_ and 5% CO_2_ at 37°C and were collected at different times. MIPRL expressions were analyzed by qRT‐PCR (*n *= 3). (C) Mouse heart tissues were isolated from the sham group or AMI group, which were further divided into non‐infarcted areas and infarcted areas. MIPRL expressions were analyzed by qRT‐PCR (*n* = 3). ns = no significant, *p < 0.05, ***p < 0.001 versus control group. AMI, acute myocardial infarction.

Hypoxia is a key injury factor for AMI. To test the potential involvement of MIPRL in hypoxia‐induced cardiac cell damage, a hypoxia model was used in human cardiomyocytes (HCM). As shown in Figure 1A, the expression of MIPRL in HCM was significantly increased at 12 and 24 h after hypoxia. We next confirmed the discovery in mouse cardiomyocytes by using the same hypoxia injury model. We found that MIPRL expression was also increased in mouse cardiomyocytes in a hypoxia‐time‐dependent manner (Figure 1B).

To provide a direct link between MIPRL and AMI, we determined and compared the expression levels of MIPRL in infarcted area, and non‐infarcted area of mouse hearts after AMI and in sham‐opened mouse hearts. Clearly, the expression of MIPRL in the infarcted area was significantly upregulated compared with that in the non‐infarcted area and in sham‐opened hearts (Figure 1C and Figure S2A).

In addition, we also detected the expression of MIPRL in other cells in the heart. The cardiac fibroblasts (Procell, CP‐M074) and mouse cardiac microvascular endothelial cells (CM‐M129, Procell) isolated from mouse hearts were used. We found that MIPRL was expressed in cardiac fibroblasts and its expression was also increased by hypoxia (Figure S2C). However, both under normal oxygen and hypoxia conditions, MIPRL was not expressed in cardiac microvascular endothelial cells (Figure S2B).

### 2.2 MIPRL has a protective role on cardiomyocytes after AMI

**FIGURE 2:**
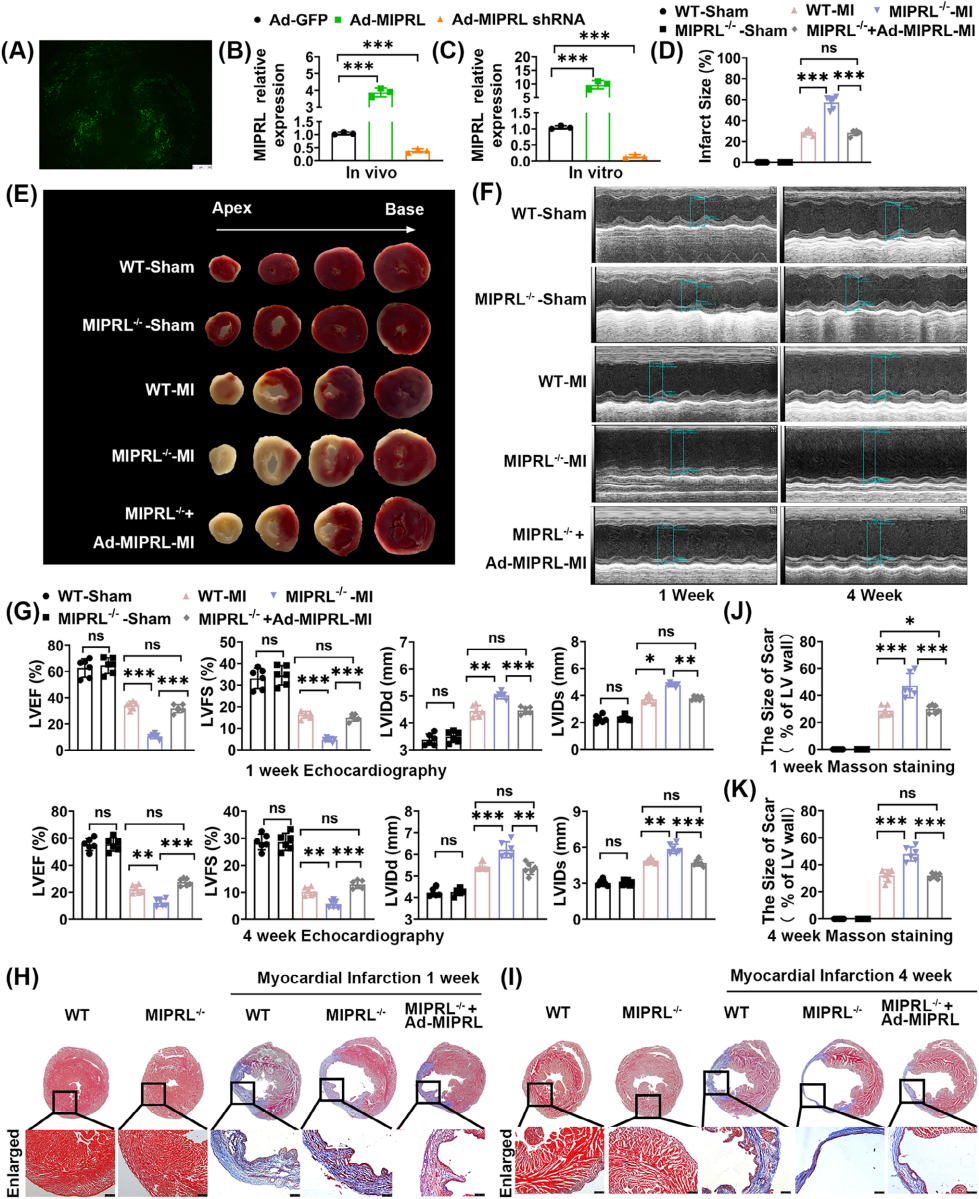
The protective effect of **m**yocardial **i**nfarction **p**rotection‐**r**elated **l**ncRNA (MIPRL) on cardiomyocytes after AMI. (A) Fluorescence image of GFP expression in cardiac tissue at 3 days after injection. (B, C) The expression of MIPRL was analyzed by quantitative real‐time polymerase chain reaction (qRT‐PCR) in heart tissues after being injected with adenovirus (B, *n* = 3) and in cardiomyocytes after being transfected with adenovirus(C, *n* = 3). (D, E) Myocardial infarction area was assessed by TTC staining at 24 h after ligation of the LAD. The red area represents viable myocardium, and the white area represents infarcted myocardium (E). Infarct areas were quantified by planimetry and expressed as a percentage of infarct area (D, *n* = 6). (F) Cardiac function was assessed by echocardiography at 1 week (Left) and 4 weeks (Right) after AMI. (G) The levels of LVEF, LVFS, LVIDd, and LVIDs in each group (*n* = 6). (H–K) Cardiac fibrosis was assessed by masson staining and the scar size was calculated as percentage of circumference of the infarct region in entire LV wall circumference (*n* = 6). Representative images of masson staining from at 1 week (H) and 4 weeks (I) after AMI. The scar sizes in each group at 1 week (J) and 4 weeks (K) after AMI. *p < 0.05, **p < 0.01, ***p < 0.001 versus control group. AMI, acute myocardial infarction; LAD, left coronary anterior descending branch; LVEF, left ventricular ejection fraction; LVFS, left ventricular fraction shortening; LVIDd, left ventricular internal diameter at end‐diastole; LVIDs, left ventricular internal diameter at end‐systole.

To determine the role of MIPRL in AMI, we generated the MIPRL knockout mice (MIPRL^−/‐^ mice)using the CRISPR/Cas 9 technology (Figure S3). We measured the effects of MIPRL deficiency on infarction size, cardiac function, and cardiac fibrosis by using these MIPRL^−/−^ mice. 2,3,5‐Triphenyltetrazolium chloride (TTC) staining showed that the myocardial infarction area in MIPRL^−/−^ mice was much larger than that in wild‐type control mice (Figure 2D,E). For the cardiac function, left‐ventricle ejection fraction (LVEF) and left‐ventricle fraction shortening (LVFS) were decreased, whereas LVIDd and LVIDs were increased in the MIPRL^−/−^ group compared with the wild‐type control group at 1 week after AMI (Figure 2F). At 4 weeks after AMI, LVEF and LVSF in the MIPRL^−/‐^ group were still significantly lower than the wild‐type control group (Figure 2F). Masson staining revealed more pronounced fibrosis in MIPRL^−/‐^ mice than in wild‐type control mice (Figure 2H–K). The results revealed that the cardiac function and structure damages were significantly aggravated by MIPRL deficiency.

To further confirm the role of MIPRL in AMI, we designed a rescue experiment by using the adenovirus expressing MIPRL (Ad‐MIPRL) in these MIPRL knockout mice. The successful up‐regulation of MIPRL via Ad‐MIPRL in heart tissue was shown in Figure 2A,C. We found both MIPRL deficiency‐exacerbated damages on cardiac function and cardiac fibrosis were inhibited by MIPRL re‐expressing via Ad‐MIPRL (Figure 2D,K).

### 2.3 MIPRL reduces the apoptosis of cardiac cells

**FIGURE 3:**
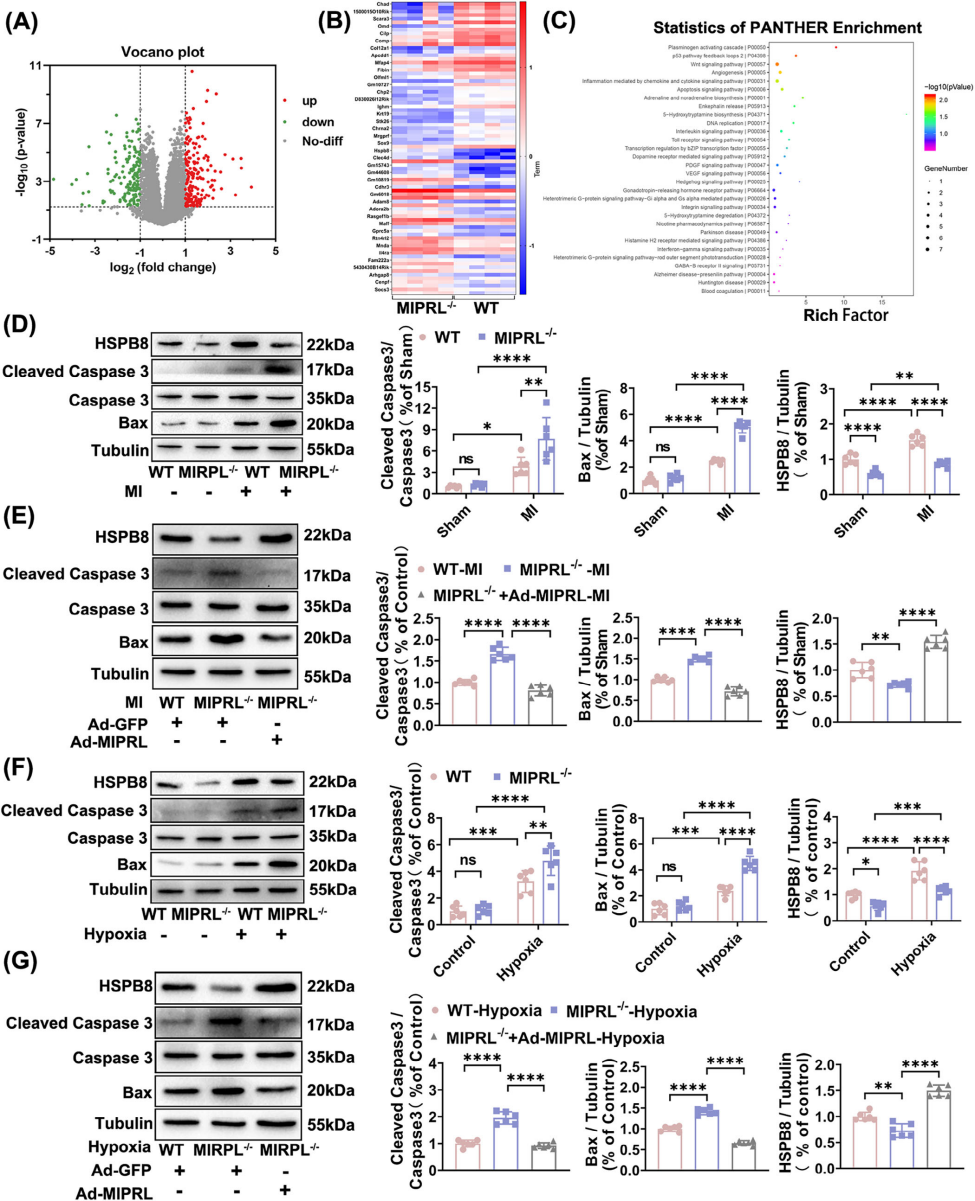
The effect of **m**yocardial **i**nfarction **p**rotection‐**r**elated **l**ncRNA (MIPRL) on apoptosis‐related signaling molecules in mouse hearts and mouse cardiomyocytes. (A) The volcano plot of all differentially expressed messenger RNA (mRNA) between wild‐type (WT) and MIPRL‐knockout mouse (MIPRL^−/‐ ^) hearts at 24 h after acute myocardial infarction (AMI) (*n* = 4 mice/group). Red and green dots represent up‐ and down‐regulated mRNA respectively. (B) Heatmap of differentially expressed mRNA. Red represents a relatively high concentration, and blue represents a relatively low concentration. (C) PANTHER enrichment of the MIPRL knockout group relative to the control group after AMI. (D) Knockout of MIPRL aggravated the expression of CC3 and Bax induced by AMI. Mice in the MIPRL‐knockout group and WT group were subjected to left coronary anterior descending branch (LAD) ligation. The expression of CC3 and Bax in heart tissue was analyzed at 24 h after AMI (*n* = 6). (E) Re‐expression of MIPRL reversed the increased expression of CC3 and Bax in MIPRL‐knockout mice. Mice were injected with adenovirus Ad‐GFP or Ad‐MIPRL and were then subjected to LAD ligation at 72 h after intramyocardial injection. The expression of CC3 and Bax in heart tissue was analyzed at 24 h after AMI (*n* = 6). (F) Cardiomyocytes isolated from WT and MIPRL‐knockout mice were exposed to hypoxia. The expression of CC3 and Bax were analyzed at 24 h after hypoxia injury (*n* = 6). (G) Cardiomyocytes isolated from WT and MIPRL‐knockout mice were transfected with Ad‐GFP or Ad‐MIPRL and then were exposed to hypoxia at 72 h after infection. The expression of CC3 and Bax were analyzed at 24 h after hypoxia injury (*n* = 6).  ns = no significant, *p < 0.05, **p < 0.01, ***p < 0.001 versus Sham group or control group. CC3, cleaved caspase‐3; WT, wild type.

Ischemia/hypoxia‐induced apoptosis is one of the key cellular mechanisms of cardiac damage in the development of AMI.^10^ We thus determined the effect of MIPRL on apoptosis of cardiomyocytes. First, we determined RNA‐sequencing in the hearts of wild‐type mice and MIPRL^−/‐^ mice after AMI for the gene‐set enrichment analysis (Figure 3A,B). According to the statistics of Panther enrichment, the apoptosis signaling pathway was one of the significant pathways (Figure 3C). Then, we determined the expression levels of apoptosis‐related signaling molecules: cleaved caspase‐3 and Bax in mouse heart tissues. We found that the expression of cleaved caspase‐3 and Bax was increased in mouse hearts after AMI (Figure 3D). MIPRL knockout elicited an additional increase in the levels of cleaved caspase‐3 and Bax in the infarcted mouse hearts (Figure 3D), which were inhibited by MIPRL re‐expression via Ad‐MIPRL (Figure 3E).

To verify the results about the effect of MIPRL on the apoptosis‐related signaling molecules in cardiomyocytes, we isolated and cultured cardiomyocytes from MIPRL^−/‐^ mice and wild‐type control mice. The expression of the cleaved caspase‐3 and Bax was determined in these cardiomyocytes with or without hypoxia injury. We found that hypoxia enhanced the expression of both cleaved caspase‐3 and Bax, while MIPRL knockout had given an additional increase in the expression of these apoptosis‐related signaling molecules (Figure 3F). In addition, the increased expression of the cleaved caspase‐3 and Bax in MIPRL‐knockout cells was significantly reversed by MIPRL re‐expression via Ad‐MIPRL (Figure 3G).

**FIGURE 4:**
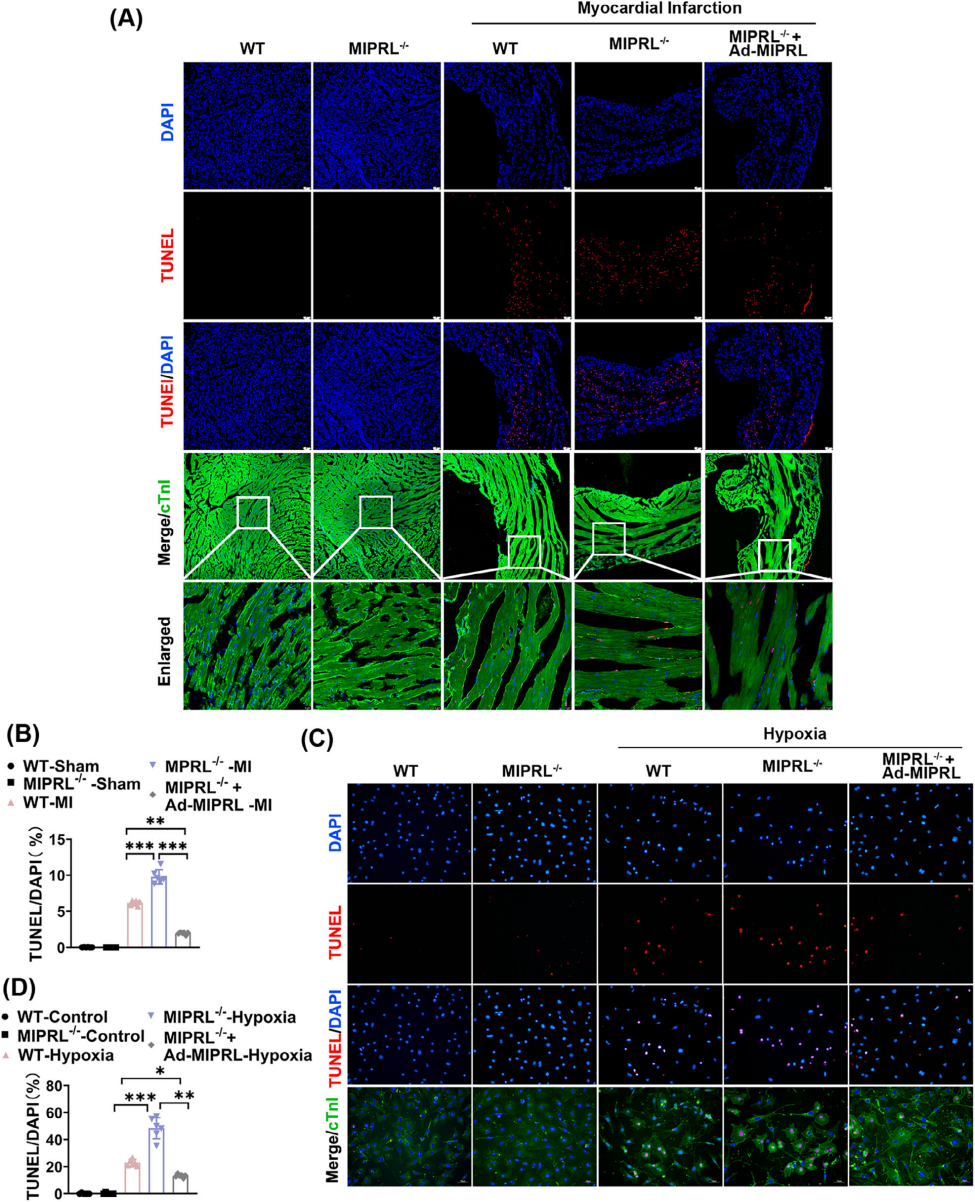
The effect of **m**yocardial **i**nfarction **p**rotection‐**r**elated **l**ncRNA (MIPRL) on apoptosis in mouse hearts and mouse cardiomyocytes. (A, B) Wild‐type (WT) and MIPRL‐knockout Mice (MIPRL^−/‐ ^) were injected with adenovirus Ad‐GFP or Ad‐MIPRL, and were then subjected to left coronary anterior descending branch (LAD) ligation at 72 h after intramyocardial injection. TUNEL staining was performed to assess the apoptotic cell induced by acute myocardial infarction (AMI). Representative photos of TUNEL were shown in (A), and quantification of the apoptosis rate was shown in (B) (*n* = 6). (C, D) Cardiomyocytes isolated from WT and MIPRL‐knockout (MIPRL^−/‐ ^) mice were transfected with Ad‐GFP or Ad‐MIPRL and then were exposed to hypoxia at 72 h after infection. Representative photos of TUNEL were shown in (C), and quantification of the apoptosis rate was shown in (D) (*n* = 6). *p < 0.05, **p < 0.01, ***p < 0.001 versus control group.

To provide direct evidence about the effect of MIPRL on cardiac cell apoptosis, we measured the rate of apoptotic cells both in mouse hearts in vivo and in cultured cardiomyocytes in vitro by TUNEL staining. The results showed that MIPRL knockout increased the cell apoptosis in mouse hearts after AMI (Figure 4A,B) and in cultured cardiomyocytes after hypoxia injury (Figure 4C,D), which were all reversed by MIPRL re‐expressing via Ad‐MIPRL (Figure 4C,D).

It is well known, that myocardial infarction could initiate various types of cell death, in addition to apoptosis, such as autophagy, and necrosis.^11^, ^12^ We thus also tested whether MIPRL had a regulatory effect on autophagy and necrosis in cardiomyocytes after hypoxia injury. The results displayed that the autophagosomes were increased in MIPRL‐deficient cardiomyocytes, but the increased autophagosomes were inhibited after MIPRL re‐expression via Ad‐MIPRL (Figure S4A–C). Meanwhile, MIPRL also had a protective effect on hypoxia‐induced myocardial necrosis as shown in Figure S4D, which displayed that the number of cardiomyocyte necrosis was decreased after MIPRL overexpression.

### 2.4 Heat shock protein B8 is involved in the anti‐apoptotic effect of MIPRL on cardiomyocytes

**FIGURE 5:**
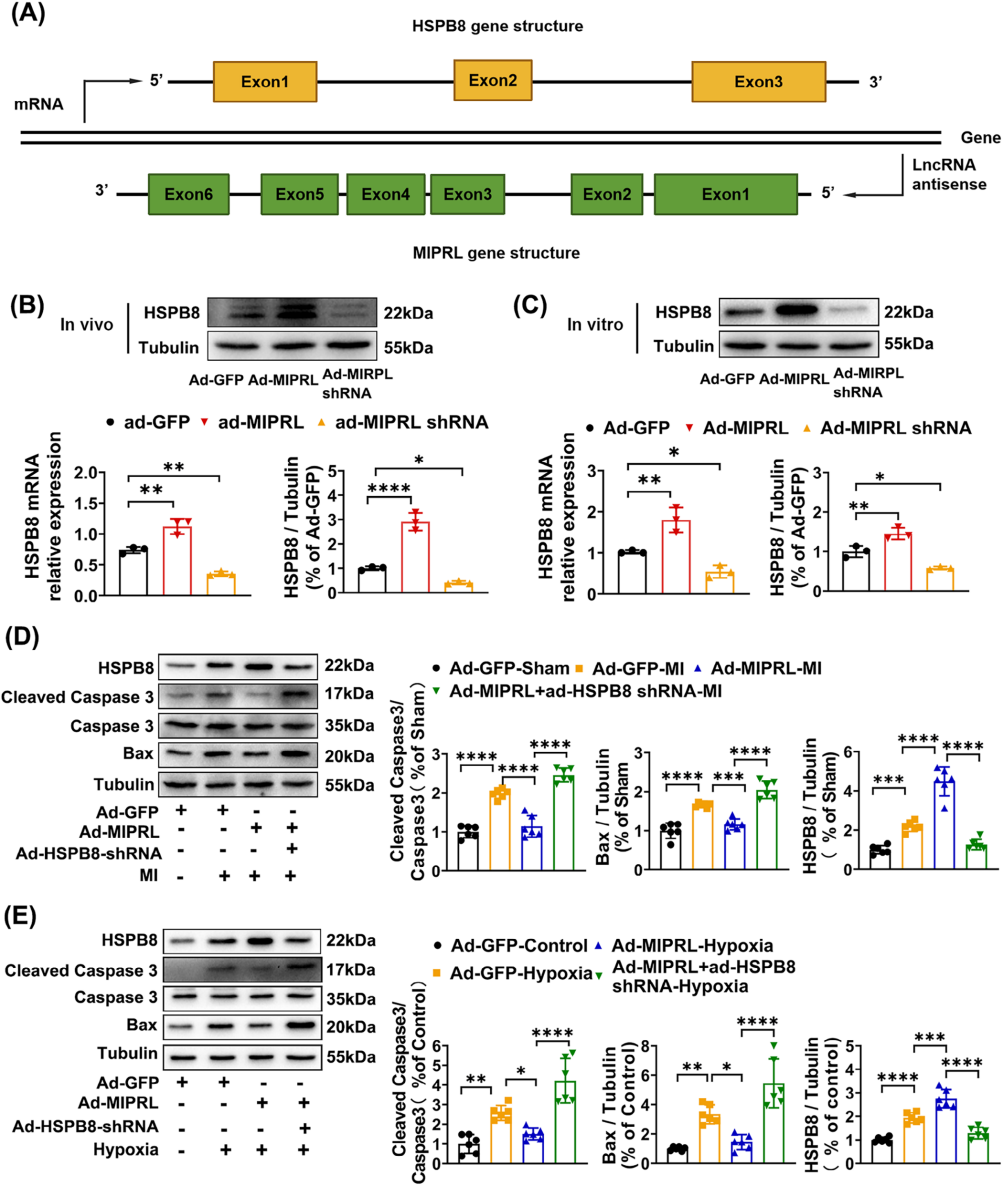
Heat shock protein beta‐8 (HSPB8) is involved in **m**yocardial **i**nfarction **p**rotection‐**r**elated **l**ncRNA (MIPRL)‐mediated effect apoptosis‐related signaling molecules in mouse hearts and mouse cardiomyocytes. (A) Schematic diagram of gene structures of MIPRL and HSPB8. (B, C) The effect of MIPRL on the expression of HSPB8. Mice were injected with Ad‐GFP, Ad‐MIPRL, or Ad‐MIPRL shRNA. The expression of HSPB8 was analyzed at 72 h after injection by quantitative real‐time polymerase chain reaction (qRT‐PCR) (left) and Western Blot (right) in (B). Cardiomyocytes isolated from neonatal mice were transfected with Ad‐GFP, Ad‐MIPRL, or Ad‐MIPRL shRNA. The expression of HSPB8 was analyzed at 72 h after infection by qRT‐PCR (left) and Western Blot (right) in (C). (D) Mice were injected with Ad‐GFP, Ad‐MIPRL, or Ad‐HSPB8 shRNA, and then were subjected to left coronary anterior descending branch (LAD) ligation 72 h after intramyocardial injection. The expression of CC3 and Bax in heart tissues was analyzed 24 h after AMI (*n* = 6). (E) Neonatal mouse cardiomyocytes were transfected with Ad‐GFP, Ad‐MIPRL, or Ad‐MIPRL shRNA, and were then exposed to hypoxia 72 h after infection. The expression of CC3 and Bax was analyzed 24 h after hypoxia injury (*n* = 6). *p < 0.05, **p < 0.01, ***p < 0.001 versus Sham group or control group.

Heat shock protein B8 (HSPB8) belongs to the small heat shock proteins related to AMI.^13^, ^14^, ^15^, ^16^ Interestingly, HSPB8 is exactly encoded by the anti‐sense strand of MIPRL (Figure 5A). Therefore, we hypothesized that HSPB8 might be involved in MIPRL‐mediated cardioprotective effects after AMI. To test this hypothesis, we determined the effect of MIPRL on the expression of HSPB8 in cultured cardiomyocytes in vitro (Figure 5B) and in mouse hearts in vivo (Figure 5C). The results showed that both in vitro and in vivo, the expression of HSPB8 was increased by MIPRL overexpression via Ad‐MIPRL. In contrast, the expression of HSPB8 was decreased in mouse hearts and in cardiomyocytes by MIPRL knockdown via Ad‐MIPRL shRNA. Thus, MIPRL is a positive regulator for the expression of HSPB8. In addition, we also found that HSPB8 itself had an anti‐apoptotic effect on cardiomyocytes with hypoxia‐injury (Figure S5) and on mouse hearts with AMI (Figure S6).

**FIGURE 6:**
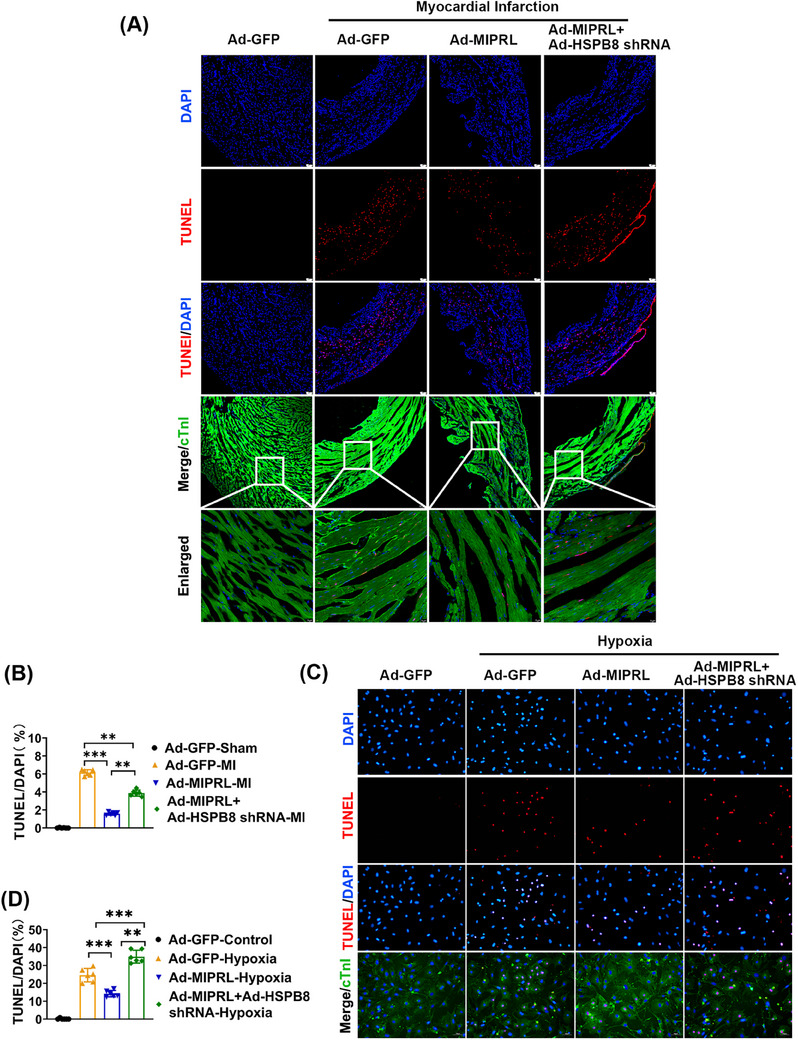
Heat shock protein beta‐8 (HSPB8) is involved in **m**yocardial **i**nfarction **p**rotection‐**r**elated **l**ncRNA (MIPRL)‐mediated effect on myocardial apoptosis. (A, B) Mice were injected with Ad‐GFP, Ad‐MIPRL, or Ad‐HSPB8 shRNA, and then were subjected to left coronary anterior descending branch (LAD) ligation 72 h after intramyocardial injection. Representative photos of TUNEL were shown in (A), and quantification of the apoptosis rate was shown in (B) (*n* = 6). (C, D) Neonatal mouse cardiomyocytes were transfected with Ad‐GFP, Ad‐MIPRL, or Ad‐MIPRL shRNA, and were then exposed to hypoxia 72 h after infection. Representative photos of TUNEL were shown in (C), and quantification of the apoptosis rate was shown in (D) (*n* = 6). *p < 0.05, **p < 0.01, ***p < 0.001 versus Sham group or control group.

To explore whether MIPRL could protect against the apoptosis of cardiomyocytes by regulating HSPB8, we designed the following experiments. As shown in Figure 6D,E, MIPRL overexpression via Ad‐MIPRL had a protective effect on the apoptosis‐related signaling molecules, whereas the effect was inhibited by HSPB8 knockdown via Ad‐HSPB8 shRNA both in mouse hearts after AMI (Figure 5D) and in cardiomyocytes with hypoxia injury (Figure 5E). Similarly, the MIPRL‐mediated protective effect on cardiac cell apoptosis was also inhibited by HSPB8 knockdown both in mouse hearts after AMI (Figure 6A,B) and in cardiomyocytes with hypoxia injury (Figure 6C,D).

**FIGURE 7:**
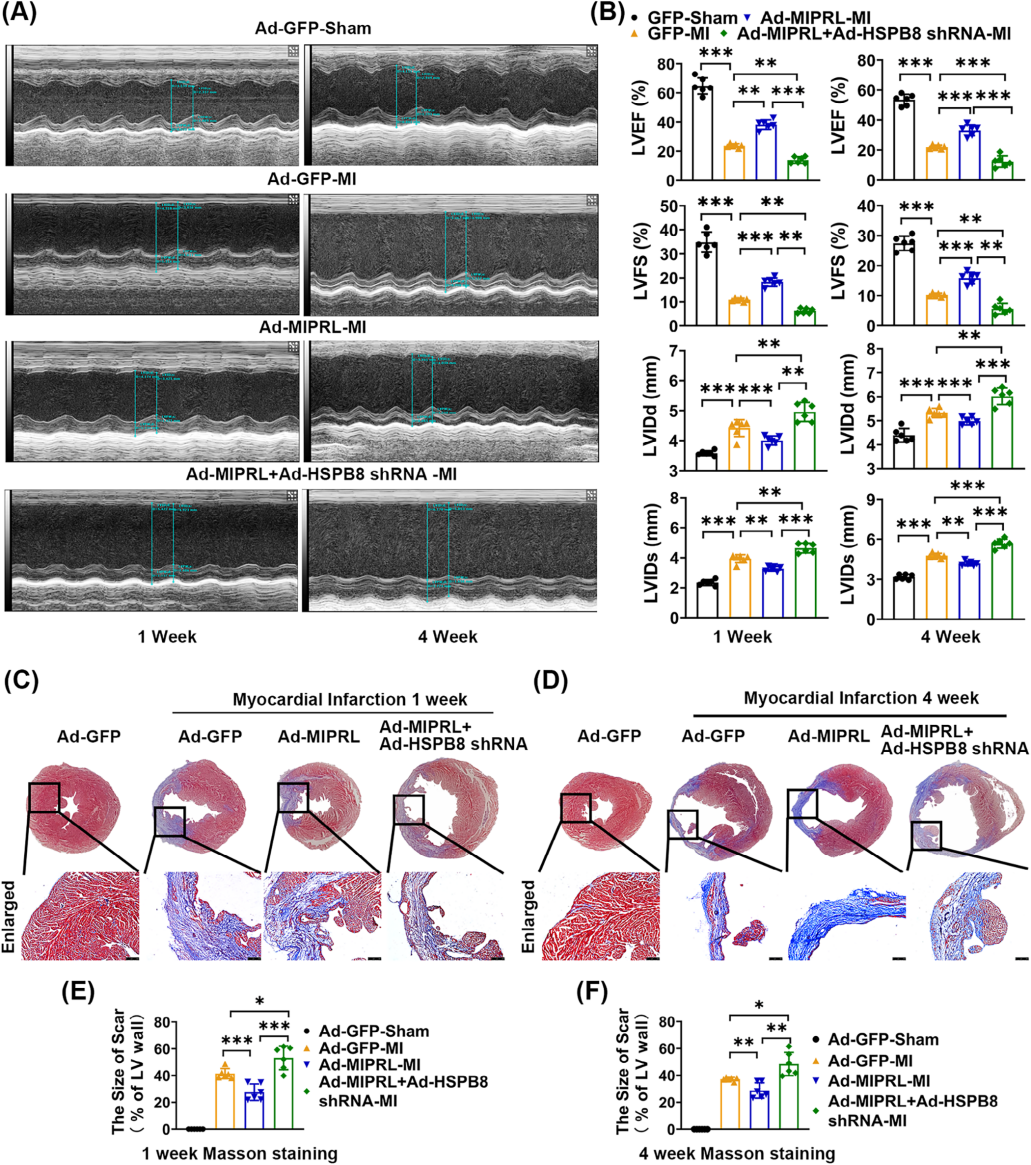
Heat shock protein beta‐8 (HSPB8) is involved in **m**yocardial **i**nfarction **p**rotection‐**r**elated **l**ncRNA (MIPRL)‐mediated cardioprotective effect on acute myocardial infarction (AMI). (A) Mice were treated with adenovirus Ad‐GFP, Ad‐MIPRL, or adenovirus Ad‐MIPRL and Ad‐HSPB8 shRNA. Cardiac function was assessed by echocardiography at 1 week (Left) and 4 weeks (Right) after AMI. (B) The levels of LVEF, LVFS, LVIDd, and LVIDs in each group (*n* = 6). (C–F) Cardiac fibrosis was assessed by masson staining and the scar size was calculated as percentage of circumference of the infarct region in entire LV wall circumference (*n* = 6). Representative images of masson staining from at 1 week (C) and 4 weeks (D) after AMI. The scar sizes in each group at 1 week (E) and 4 weeks (F) after AMI. *p < 0.05, **p < 0.01, ***p < 0.001 versus control group. LVEF, left ventricular ejection fractions; LVFS, left ventricular fraction shortening; LVIDd, left ventricular internal diameter at end‐diastole; LVIDs, left ventricular internal diameter at end‐systole.

Subsequently, we determined the potential involvement of HSPB8 in the MIPRL‐mediated cardioprotective effect in the mouse AMI model. Echocardiography was performed to evaluate the cardiac function at 1 week and 4 weeks after AMI. As shown in Figure 7A,B, LVEF, and LVSF were increased with MIPRL overexpression but were decreased with HSPB8 knockdown. In contrast, LVIDd and LVIDs were decreased with MIPRL overexpression but were increased with HSPB8 knockdown at both 1 week and 4 weeks post AMI. In addition, the Masson staining showed that in mice with AMI, the MIPRL overexpression attenuated, whereas HSPB8 knockdown aggravated the cardiac fibrosis compared to that of the control mice at 1 week (Figure 7C, E) and 4 weeks (Figure 7D, F) after AMI. Thus, the MIPRL overexpression‐mediated cardioprotective effects on AMI were effectively inhibited by down‐regulation of HSPB8.

### 2.5 MIPRL increases the stability of HSPB8 messenger RNA

**FIGURE 8:**
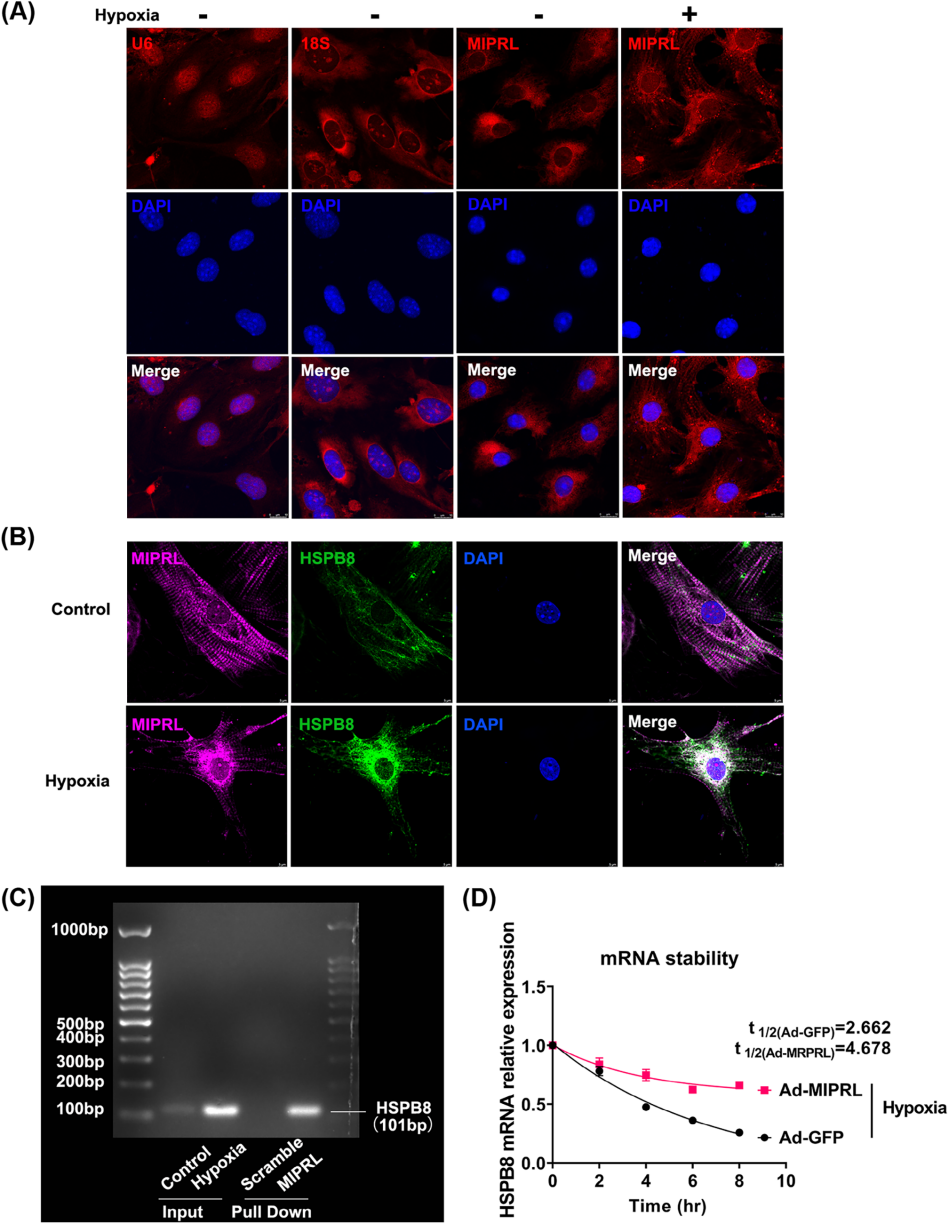
**M**yocardial **i**nfarction **p**rotection‐**r**elated **l**ncRNA (MIPRL) increases the stability of heat shock protein beta‐8 (HSPB8) messenger RNA (mRNA). (A) RNA fluorescence in situ hybridization (RNA‐FISH) assay on neonatal mouse cardiomyocytes showed that MIPRL was localized to the cytoplasm. U6, located in the nucleus, and 18S, located in the cytoplasm were used as internal reference. (B)The co‐localization of MIPRL and HSPB8 mRNA was assessed by double RNA‐FISH assay in cardiomyocytes after hypoxia. Probes were constructed against MIPRL labeled with Cy3 dye (red) and HSPB8 mRNA labeled with FAM dye (green). (C) The binding of MIPRL and HSPB8 mRNA was assessed by RNA‐RNA pull‐down assay. Cardiomyocytes were exposed to hypoxia for 24 h. Specific probes with a strong affinity were constructed against different regions of MIPRL and were then added to the cardiomyocyte extracts. Non‐specific probes were added as the control group. A representative image of DNA gel electrophoresis is shown in (C). (D) The increase of stability in HSPB8 mRNA by MIPRL was assessed via mRNA stability assay. Cardiomyocytes were transfected with Ad‐GFP or Ad‐MIPRL, and were then exposed to hypoxia range from 2 to 8 h. Actinomycin D (10 μg/mL) was added to the medium before hypoxia. Decay curves of HSPB8 mRNA were generated by GraphPad Prism.

Our results above suggested that MIPRL has a regulatory effect on the expression of HSPB8 messenger RNA (mRNA). However, the detailed molecular mechanism was still unknown. We first performed the RNA fluorescence in situ hybridization (RNA‐FISH) assay to test the subcellular location of MIPRL. As shown in Figure 8A, MIPRL was localized to the cytoplasm and was more accumulated in the cytoplasm after hypoxia injury.

It is reported that the sense RNA and antisense lncRNA transcripts can hybridize and form RNA duplexes which modulate sense mRNA expression.^17^ In addition, nuclear and cytoplasmic sense‐antisense hybrids can alter mRNA stability and modulate translation.^17^, ^18^ Considering that MIPRL, as an antisense lncRNA, was localized in the cytoplasm and has a positive regulatory effect on HSPB8 mRNA, we thus speculated that MIPRL might increase the HSPB8 mRNA stability by combining with HSPB8 mRNA. To test this, we determined a double RNA‐Fish assay to reveal the colocalization of MIPRL and HSPB8 mRNA. As shown in Figure 8B, there was a fluorescence overlap between MIPRL and HSPB8 mRNA in cardiomyocytes after hypoxia injury. We then demonstrated that MIPRL could combine with HSPB mRNA by RNA‐RNA pull‐down assay. Indeed, HSPB8 mRNA was enriched after the MIRPL pull‐down was performed with its specific probes in mouse cardiomyocyte extracts (Figure 8C). We further determined whether MIPRL could increase HSPB8 mRNA stability. Using transcription inhibition by Actinomycin D, it was clear that HSPB8 mRNA in MIPRL over‐expressed cardiomyocytes decayed more slowly than that in the control cells under the hypoxic condition (Figure 8D). Similarly, under normoxia, the degradation rate of HSPB8 mRNA in cardiomyocytes overexpressing MIPRL was also decreased compared to control cells with normal expressing levels of MIPRL (Figure S7).

## 3 DISCUSSION

lncRNAs have been found to be aberrantly expressed in many diseases. Among them, some lncRNAs may have protective effects against diseases, whereas others may have pro‐disease effects. For example, lncRNA ANRIL can increase the risk of coronary heart disease and type II diabetes, and its expression level is associated with the risk of atherosclerosis.^19^ lncRNA MALAT1 is reported to be able to enhance endothelial cell inflammation and oxidative stress damage by regulating SAA3 expression on high glucose‐induced injury in human umbilical vein endothelial cell.^20^ lncRNA APF likewise promotes the expression of ATG7 by directly binding to miR‐188‐3p and blocking the activity and then aggravates the damage of myocardial autophagy.^21^ ^^lncRNAs are now becoming the promising molecular targets for the new drug development to treat and prevent many human diseases by using preclinical animal models.

It is well‐known that apoptosis is one of the key cellular events in the pathogenesis of AMI.^22^, ^23^, ^24^ To date, an increasing number of lncRNAs are found to be key regulators of various cellular processes including apoptosis.^25^, ^26^, ^27^, ^28^, ^29^ MIPRL is a newly identified lncRNA. Previous studies have only shown that MIPRL is expressed in human hearts and its expression is increased in human heart tissues after ischemia.^7^ However, its detailed function in cardiomyocytes and in hearts remains unknown. Here, we have identified for the first time that MIPRL exerts the cardioprotective effect by alleviating cardiac cell apoptosis in hearts after AMI.

Unlike other non‐coding RNAs such as miRNAs, lncRNAs are poorly conserved among species. By using Clustal Omega which is a multiple sequence program to generate alignments between human‐derived and mouse‐derived lncRNAs,^9^ we found there were multiple conserved residues between human and mouse MIPRL sequences. It should be noted that, unlike small ncRNAs such as miRNAs, lncRNAs often have multiple functional residues, which suggests that the conservation of lncRNAs among different species might not require the same sequence in the whole lncRNA molecule. We then constructed a MIPRL knockout mouse and determined the effects of MIPRL deficiency on infarction size, cardiac fibrosis, and cardiac function after AMI. Clearly, the damages of AMI were aggravated when MIPRL was deficient. The above adverse results of MIPRL deficiency could be reversed by MIPRL re‐expression via intramyocardial injecting of Ad‐MIPRL. The results have revealed that MIPRL has a protective effect against AMI.

HSPB8 is a member of the small heat shock protein superfamily, which is widely distributed and is involved in regulating a variety of physiological and biochemical processes in the body. It is well established that HSPB8 has an anti‐apoptotic effect in different diseases. For example, HSPB8 is able to ameliorate the lipopolysaccharide‐induced myocardial injury by inhibiting apoptosis.^30^ Moreover, HSPB8 interacts with other small HSPs, such as HSP27 and B crystalline, to promote cell survival and decrease apoptosis.^31^ In contrast, in SK‐MEL‐2, PC‐3, and TC32 cells, DNA demethylation leading to increased expression of HSPB8 or transient transfection with HSPB8 expression vector which is accompanied by caspase and p38MAPK‐dependent apoptosis.^32^ In the current study, RNA‐seq analysis and functional enrichment revealed that apoptosis was involved in the biological functions of MIPRL. Considering that HSPB8, a potential downstream target gene of MIPRL, has an important role in anti‐apoptosis, we thus focused our research on cardiac cell apoptosis. We found that the apoptosis was promoted in cardiomyocytes after hypoxia injury or in heart tissues after AMI, and was aggravated when MIPRL is deficient. The rescue experiment was performed in MIPRL‐deficient cardiomyocytes and in MIPRL‐deficient mouse hearts to determine the reversal effect of cardiac cell apoptosis by re‐expression of MIPRL. In addition, when HSPB8, the target gene of MIPRL was deficient, MIPRL‐induced anti‐apoptotic effects on cardiomyocytes and heart tissues were inhibited. These results confirmed that HSPB8 was indeed involved in the MIPRL‐mediated anti‐apoptotic effect.

HSPB8 is reported to play a protective role in different diseases by regulating different stages of autophagy in addition to apoptosis.^33^, ^34^, ^35^ For example, HSPB8 could be combined with DUSP12 and then decrease the apoptosis and oxidative stress in myocardial I/R injury via mitophagy.^36^ We thus also examined whether MIPRL had a regulatory effect on autophagy in cardiomyocytes after hypoxia injury. The results displayed that the autophagosomes were increased when MIPRL was deficient, but were inhibited after MIPRL re‐expression. In addition, MIPRL also had a protective effect on necrosis of cardiomyocytes. The effects and the detailed mechanisms of MIPRL on mitophagy and necrosis should be determined in future studies.

What was the molecular mechanism of MIPRL in regulating the HPSB8 expression? We found that MIPRL was co‐localized with HPSB8 mRNA in the cytoplasm and has a positive regulatory effect on HSPB8 mRNA. A double RNA‐Fish assay and RNA‐RNA pull‐down assay revealed that MIPRL could bind to HSPB8 mRNA and inhibit its degradation, and thus upregulate HSPB8 expression in cardiomyocytes and hearts.

## 4 CONCLUSIONS

In summary, our study reveals a significant protective effect of a novel lncRNA, named MIPRL, against AMI. The underlying mechanisms for this action might be related to its apoptotic effect by increasing the stability of HSPB8 mRNA. MIPRL might be a novel promising therapeutic target for ischemic heart diseases.

## 5 MATERIALS AND METHODS

### 5.1 Mice, intramyocardial injection, and establishment of AMI model in mice

Wild‐type mice (C57BL/6J) and MIPRL knockout mice (C57BL/6J background), male and 10–12 weeks, were purchased from Biocytogen Pharmaceuticals (Beijing). MIIPRL knockout (MIPRL^−/−^) genotype was confirmed using polymerase chain reaction (PCR) analysis of tail DNA and the expression of MIPRL in mouse hearts was analyzed using quantitative real‐time PCR (qRT‐PCR) analysis of tissue. The intramyocardial injection was performed 3 days before AMI. Under 2% isoflurane inhalation anesthesia, a small skin incision was made on the left breast of the mice to expose the heart. The needle was introduced into the blood supply area of the left coronary anterior descending branch (LAD) for the injection of adenovirus at a dose of 1.0 × 109 pfu/heart. The AMI model was induced by permanent LAD ligation in mice. In brief, mice were anesthetized with 2% isoflurane inhalation and then placed in a supine position for thoracotomy. The heart was exposed for the ligation of the LAD. Blanching was apparent at the left anterior wall and the apex of the heart to confirm the successful model.

All the animal surgeries and animal models in this study were performed under general anesthesia with 2% isoflurane inhalation. At the end of all the animal experiments, the animals were euthanized with an overdose of sodium pentobarbital (200–250 mg/kg intraperitoneal injection). The procedures of anesthesia and euthanizing method were consistent with the Guide for the Care and Use of Laboratory Animals (updated (2011) version of the NIH guidelines). All experiments in this study were approved by the Institutional Animal Use and Care Committee, at Wenzhou Medical University.

### 5.2 Isolation and culture of neonatal mouse cardiomyocytes

For neonatal cardiomyocyte isolation, we modified a previously reported protocol. ^37^ Cardiomyocytes were isolated from neonatal (1–3 days) C57BL/6J wildtype and MIPRL knockout mice. After dissection and mincing, hearts were incubated with 0.0125% trypsin (Gibco, 15050057) at 4°C overnight. Then, 1.5 mg/mL of collagenase II (Worthington, LS004176) was added to digest the tissue fragments on the shaker at 37°C. After digestion, the tissue fragments were gently triturated and centrifuged at 1000 rpm for 5 min. The cell pellet was resuspended in a plating medium (65% DMEM high glucose [Gibco,11995065], 19% DMEM/F‐12 [Gibco, 11330032], 10% horse serum [Gibco,16050122], 5% fetal bovine serum [Gibco, 10099141], and 1% penicillin/streptomycin [Gibco, 15140122]) and incubated for 1 h as a pre‐plating step. Finally, the cells were plated into 1% matrigel (Corning, 354234) coated dishes and cultured in the plating medium. On the next day, the medium was changed to the maintenance medium (78% DMEM high glucose, 17% DMEM/F‐12, 4% horse serum, 1% penicillin/streptomycin, and 0.1 mM BrdU [Sigma, B5002]). Then cultured for an additional 1 day, cells were subjected to subsequent experiments.

### 5.3 Cell Culture, adenovirus infection, and hypoxia models

Immortalized HCM (ABM, T0519) were purchased from Applied Biological Materials Inc., and cultured in Prigrow I medium (ABM, TM001) containing 10% FBS and 1% penicillin‐streptomycin. Mouse cardiac endothelial cells (Procell, CP‐M129) and Mouse cardiac fibroblasts (Procell, CP‐M074) were purchased from Procell Life Science & Technology. They were cultured with their own specialized medium (Procell, CM‐M129 for CP‐M129; CM‐M074 for CP‐M074). Neonatal mouse cardiomyocytes were extracted as described above. Cells were seeded in dishes and cultured to 70%–80% confluence. Replace the maintenance medium (DMEM with 2% FBS), and add adenovirus into the medium (MOI = 50 for neonatal mouse cardiomyocytes). Three days after the adenovirus infection, cells were cultured in a serum‐free medium in a hypoxic condition with 1% O_2_ and 5% CO_2_ ranging from 6 to 24 h.

### 5.4 Quantitative RT‐PCR

Total RNAs from cardiomyocytes or heart tissues were extracted using TRIzol (Invitrogen, 15596018) according to the manufacturer's instructions. First‐strand cDNA was synthesized with One‐Step gDNA Removal and cDNA Synthesis SuperMix (Transgen, AH311) and analyzed by real‐time quantitative PCR with Top Green qPCR SuperMix (Transgen, AQ131). The reaction mixtures containing SYBR Green were composed following the manufacturer's protocol. 18S RNA was used as the housekeeping denominator.

### 5.5 Western Blot

The cells were lysed in the RIPA buffer containing a PMSF (Beyotime, ST506) and phosphatase inhibitor (Roche, 4906845001), and were centrifuged at 12,000 rpm for 30 min at 4°C for protein isolation and collection. The protein concentrations were determined by using the BCA Protein Assay Kit (Beyotime, P0009). The samples were subjected to sodium dodecyl‐sulfate polyacrylamide gel electrophoresis and transferred to PVDF membranes (Millipore, ISEQ85R), and incubated with primary antibodies overnight at 4°C. The following antibodies were used: anti‐HSPB8 (Cell Signaling Technology, 3095), anti‐Bax (Cell Signaling Technology, 2772), anti‐Cleaved Caspase‐3 (Cell Signaling Technology, 9664), anti‐Caspase‐3 (Cell Signaling Technology, 9665), anti‐β‐Tublin (Cell Signaling Technology, 2146). The Horseradish peroxidase‐labeled secondary antibodies (ZEN BIO, 511201) were added after three times washing with TBST. After 2 h, antigen‐antibody complexes were detected using a WesternBright ECL kit (Advansta, K‐12045) and visualized by the Bio‐Rad gel imaging system. Quantification analysis was processed by Image J software.

### 5.6 TTC staining for assessment of myocardial infarction area

In brief, at 24 h after AMI, mouse hearts were quickly frozen at −20°C, and were sliced into four pieces from the apex to the base, then were incubated in 2% TTC solution (Sigma, T8877) for 30 min at 37°C. The sliced sections were fixed with 4% paraformaldehyde and photographed under a dissecting microscope.

### 5.7 Echocardiography

The echocardiographs were recorded at 1 and 4 weeks after AMI using a Vevo 1100 system equipped with a 30‐MHz transducer (Visual Sonics). LV end‐diastolic dimension (LVEDD), LV end‐systolic dimension (LVESD), LV internal diameter at end‐diastole (LVIDd), and LV internal diameter at end‐systole (LVIDs) were measured based on an M‐mode echocardiogram of the short axis of the left ventricle. The LVEF and LVFS values were calculated using computer algorithms via Teichholz's method.

### 5.8 Cardiac histology and immunofluorescence

The heart tissue was fixed with 4% paraformaldehyde, dehydrated stepwise with sucrose, and embedded in an O.C.T Compound (SAKURA, 4583). The samples were then cut into 5 μm slices on a microtome and subjected to a Masson trichrome staining kit (Solarbio, G1340) and immunofluorescence staining. Masson trichrome staining was used for evaluating collagen fiber organization and was performed according to the manufacturer's protocol. For immunofluorescence staining, the slices were fixed with 4% paraformaldehyde, permeabilized with 0.5% Triton‐100 (Solarbio, T8200) for 5 min on ice, blocked with 0.5% BSA, then stained with anti‐CD31 (Abcam, ab28364) or anti‐Cardiac Troponin I (Proteintech, 66376‐1‐Ig) overnight at 4°C. After rinsed thrice with PBST, the slices were incubated with anti‐Mouse labeling Alexa Fluor 488 for 2 h at room temperature. Following DAPI staining, the image was taken by fluorescence microscopy or confocal microscopy.

### 5.9 TUNEL assay

The cardiomyocyte apoptosis was evaluated using the Situ Cell Death Detection Kit (Roche, 12156792910) according to the manufacturer's instructions. For the cell TUNEL staining, the medium was removed and the cells were washed with PBS, and fixed with 4% paraformaldehyde. Cells were permeabilized with 0.5% Triton‐100 for 5 min on ice, then were incubated with a reaction mixture at 37°C for 1 h. DAPI was used to counterstain the nuclei. Images were captured under the microscope and the Image Lab software was used to calculate the apoptosis rate. For the tissue TUNEL staining, the heart tissue was fixed with 4% paraformaldehyde, dehydrated stepwise with sucrose, embedded, sectioned, and stained as described above.

### 5.10 RNA‐FISH assay

Cardiomyocytes were fixed with 4% paraformaldehyde for 10 min, and permeabilized with 0.5% Triton‐100 for 5 min on ice. After pre‐hybridized at 37°C for 30 min, the cells were incubated at 37°C overnight for hybridization with the probe of MIPRL containing a 5′‐Cy3 dye (Ribobio) or the probe of HSPB8 mRNA containing a 5′‐FAM dye (Genepharma). Then rinsed with different concentrations of SSC, and incubated with DAPI for 10 min, the cells were observed under a confocal microscope. For protein‐RNA Double staining (IF/FISH), the dual labeling procedure was performed as described before.^38^ In brief, the cells were fixed, permeabilized, and hybridized with a probe. Then rinsed with SSC, the cells were blocked for 1 h, incubated with primary antibody at 4°C overnight, and Alexa Fluor Secondary Antibodies at room temperature for 2 h. Following DAPI staining, the image was taken by confocal microscopy.

### 5.11 RNA‐RNA pull down

RNA‐RNA pull‐down procedure was performed as described.^40^ Cardiomyocytes were seeded in the 10 cm culture dish and transfected with Ad‐MIPRL. After 72 h, the culture medium was changed to serum‐free medium and the cells were cultured in hypoxic conditions (1% O_2_, 5% CO_2_ for 24 h. The cells were fixed with 1% paraformaldehyde, then quenched the paraformaldehyde with 1.25 M glycine. The cell pellets were collected and resuspended with the Lysis Buffer (50 mM Tris‐HCl, 10 mM EDTA, 1% SDS, 200 U/mL RNAse inhibitor solution, 5 μL/mL proteases inhibitor), and the hybridization buffer (50 mM Tris‐HCl, 750 mM NaCl, 1 mM EDTA, 1% SDS, 15% Formamide) with 100 pmol of oligonucleotide probes (specific or non‐specific; see Table S1) was added. After incubating for 6 h, magnetic streptavidin beads supplemented (Thermo Scientific, 26157) with RNase inhibitor and a cocktail of proteases inhibitor were added and were incubated overnight under moderate agitation. RNAs were separated from the beads and were purified with the purification kit (Thermo Scientific, K0842). HSPB8 mRNAs were determined by PCR followed by DNA electrophoresis.

### 5.12 mRNA stability assay

Actinomycin D is a transcription inhibitor by intercalating into DNA and is widely used in mRNA stability assays.^39^ Cardiomyocytes were seeded in a 12‐well culture plate. After 72 h, the culture medium was changed to a serum‐free medium containing 10 μg/mL actinomycin D (Selleck, S8964). Then cells were cultured in the hypoxic condition and were collected at 2, 4, 6, and 8 h time points. qRT‐PCR was performed as described above.

### 5.13 Statistical analysis

Data are expressed as the mean ± SD. At least three independent experiments for each cellular or animal experimental group. We evaluated the data with a Student's t‐test. We used a one‐way analysis of variance for multiple comparisons. The results were considered to be statistically significant when *p*< 0.05.

## AUTHOR CONTRIBUTIONS

Chunxiang Zhang is the PI of the study and directed the study. Xueqiang Guan and Rongzhou Wu also directed the study. Rongzhou Wu and Tingting Wu designed the experiments and performed most of the experiments. Qiaoyu Wang, Youyang Shi, Qianqian Dong, Xing Rong, Meiting Chen, Zhiyu He, Yu Fu, Lei Liu, and Shuai Shao performed the experiments and did data analysis. All authors have read and approved the final manuscript.

## CONFLICT OF INTEREST STATEMENT

The authors declare no conflict of interest.

## ETHICS STATEMENT

The animal experiment in this study was approved by the Institutional Animal Use and Care Committee, Wenzhou Medical University (Approval Number: wydw2022‐0307).

## Supporting information

Supporting Information

## Data Availability

Data supporting the findings of this study are available from corresponding authors upon reasonable request.
