# Supplementary material for: The ischemia‐enhanced myocardial infarction protection‐related lncRNA protects against acute myocardial infarction
Source: MedComm (2020). 2024 Jul 10;5(7):e632. doi: 10.1002/mco2.632 (PMC11234438; doi:10.1002/mco2.632)
Supplement: Supplementary file 1 — Supporting Information [file MCO2-5-e632-s001.pdf]

Ischemia-enhanced lncRNA MIPRL protects against acute myocardial infarction

Short Title: MIPRL in acute myocardial infarction

Rongzhou Wu[1#]*, Tingting Wu[1#], Qiaoyu Wang[1], Youyang Shi[1], Qianqian Dong[1], Xing Rong[1], Meiting Chen[3], Zhiyu He[3], Yu Fu[1], Lei Liu[1], Xueqiang Guan[1]*, Chunxiang Zhang[2, 1]*

[1]Children's Heart Center, The Second Affiliated Hospital and Yuying Children's Hospital of Wenzhou Medical University, Institute of Cardiovascular Development and Translational Medicine, The Second School of Medicine, Wenzhou Medical University, Wenzhou, Zhejiang 325027, China

[2]Department of Cardiology, Key Laboratory of Medical Electrophysiology, Ministry of Education, Institute of Cardiovascular Research, Institute of Metabolic Diseases, the Affiliated Hospital of Southwest Medical University, Southwest Medical University, Luzhou, Sichuan, 646000, China.

[3]The University of Alabama at Birmingham, Birmingham, Alabama 35294, USA

*Correspondence: Chunxiang Zhang, E-mail: zhangchx999@163.com；Xueqiang Guan, E-mail: wzsgxq@163.com；Rongzhou Wu, E-mail: wrz71@hotmail.com

[#]Rongzhou Wu and Tingting Wu contributed equally to this work.

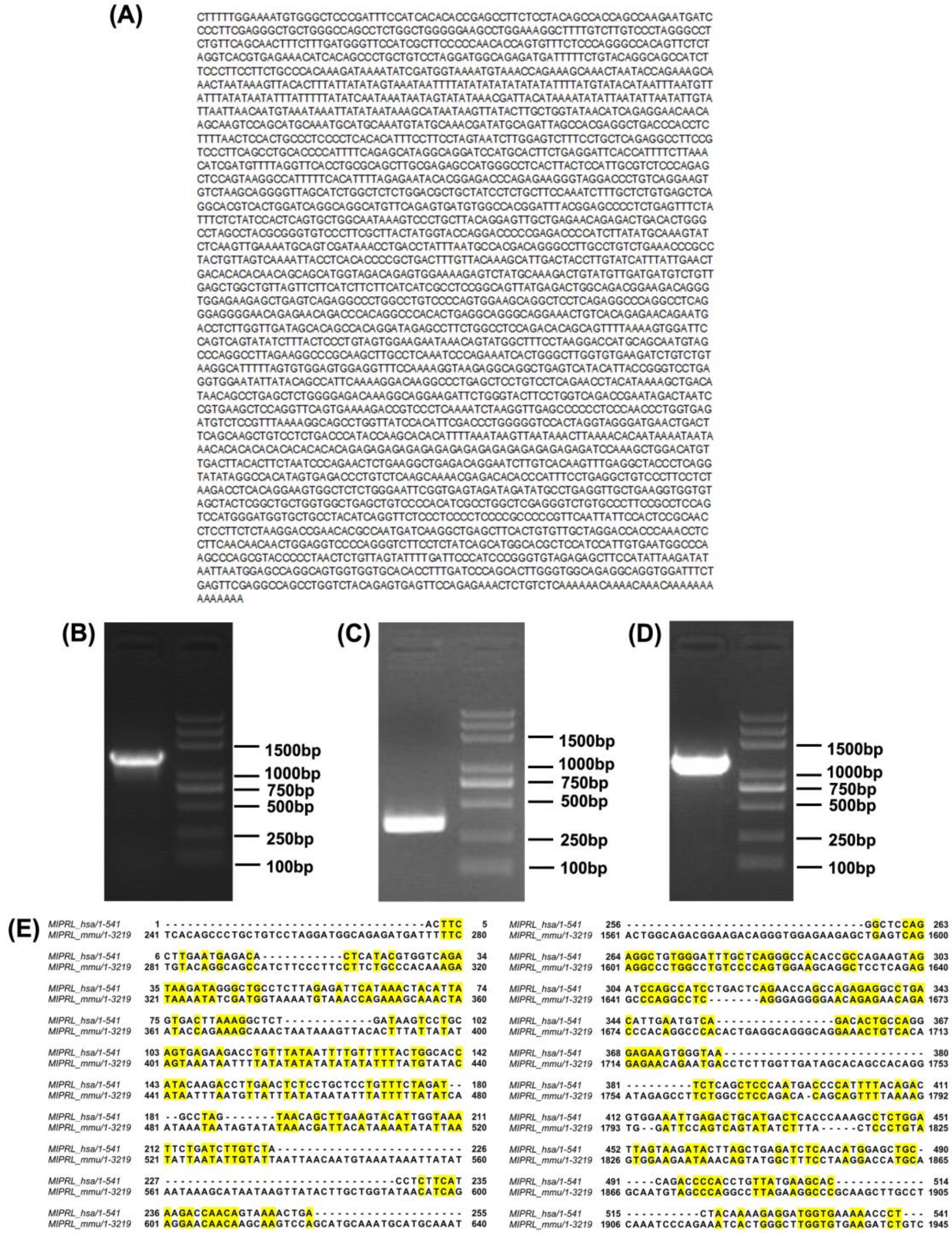

Figure S1. The homology between human and mouse MIPRL. (A)Sequencing analysis of mouse MIPRL by RACE. (B)Representative gel electropherogram of 5'-RACE PCR product. (C)Representative gel electropherogram of 3'-RACE PCR product. (D)Representative gel electropherogram of intermediate genes product. (E)The homology between human and mouse MIPRL was assessed by Clustal Omega, a multiple sequence program to generate alignments. Fully conserved residues between the two sequences were highlighted in yellow.

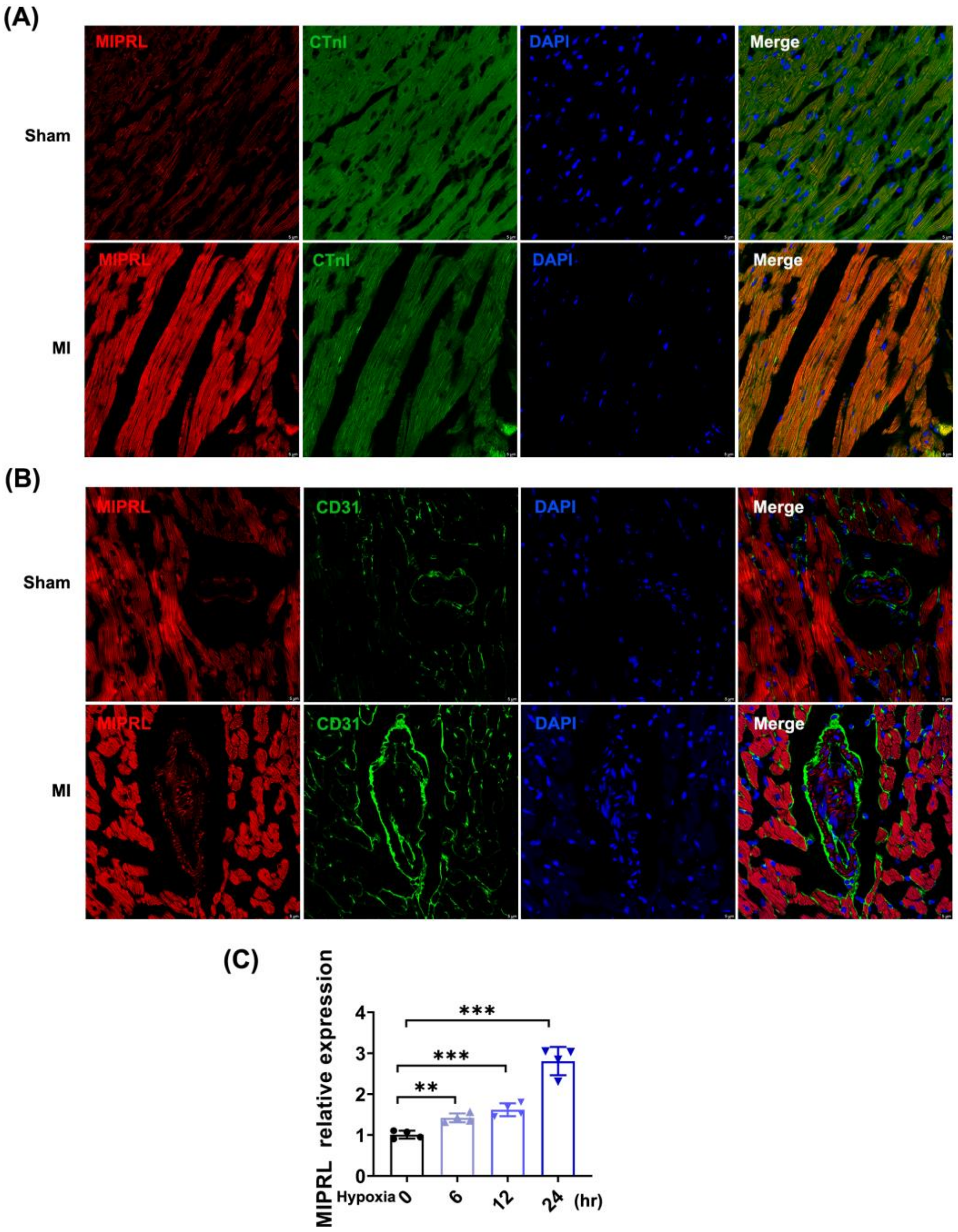

Figure S2. The expression of MIPRL in different cells after AMI or hypoxia injury. (A-B) Immunofluorescence/RNA-FISH staining shown the expression and location of MIPRL in cardiomyocytes (A) and endothelial cells (B) at 72h after AMI. MIPRL probe was labeled with Cy3 dye (red). Both cardiac troponin I (CTnI) representing cardiomyocyte in (A), and CD31 representing endothelial cells in (B) were labeled with Alexa Fluor 488 dye (green). (C)Cardiac fibroblasts were given hypoxia injury with 1% O_2 and 5% CO_2 at 37°C. Then cells were collected at different times and MIPRL expression was analyzed by qRT-PCR (n=4). $^{**}P<0.01$, $^{***}P<0.001$ vs. control group.

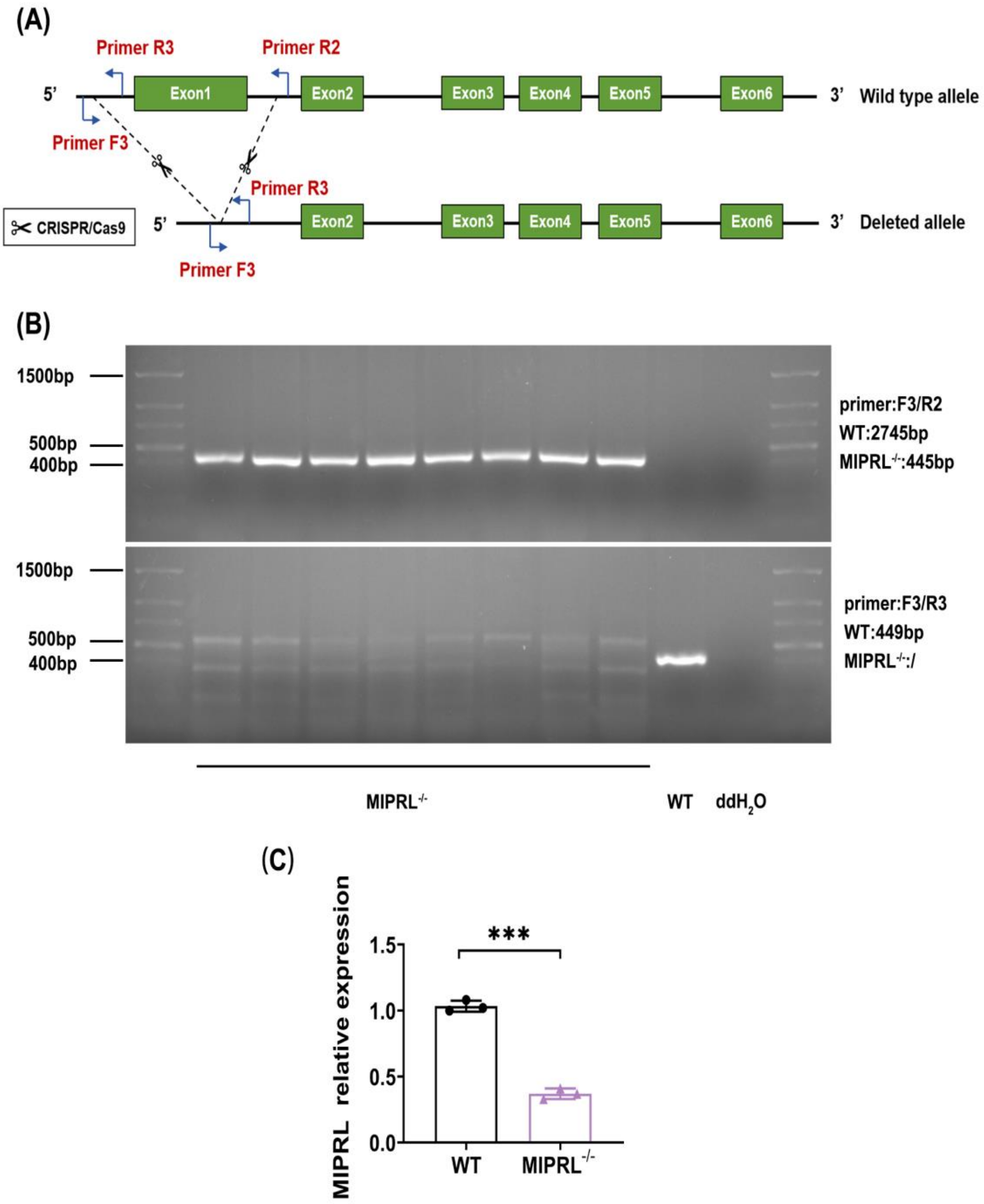

Figure S3. Construction and validation of MIPRL knockout mice. (A)The strategy of gene knockout in MIPRL-knockout mouse with CRISPR/Cas9 technology. Primer binding sites were shown in diagram. (B)Results of PCR gel electrophoresis of mouse tail DNA of homozygous mouse. (C)The expression of MIPRL in heart tissue from wild-type or MIPRL-knockout mouse (n=3). ***P<0.001 vs. wild-type group.

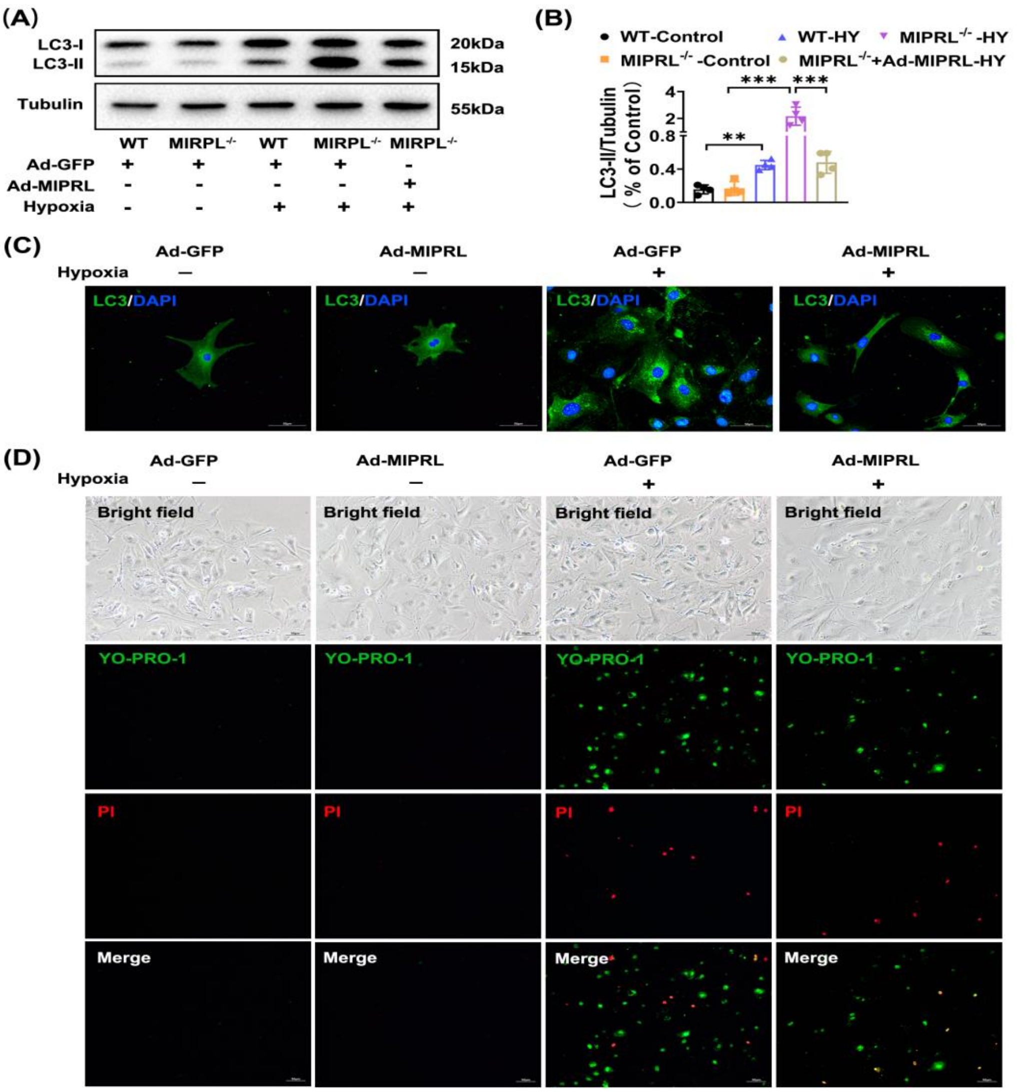

Figure S4. The effect of MIPRL on autophagy and necrosis in cardiomyocytes. (A-B) The protective effect of MIPRL on autophagy was assessed by Western Blot. Cardiomyocytes isolated from WT or MIPRL-knockout mice were transfected with Ad-GFP or Ad-MIPRL, then were exposed to hypoxia 72h after infection. The expression of LC3 was analyzed 24h after hypoxia injury (n=4). Representative images of immunoblotting were shown in (A). The densitometric analysis of western blot of LC3-II was shown in (B). (C)Autophagic vacuoles were examined by immunofluorescence staining. Representative photos of LC3 puncta (green) were shown. (D)The protective effect of MIPRL on necrosis was assessed by apoptosis and necrosis detection Kit. Cardiomyocytes were transfected with Ad-GFP or Ad-MIPRL, then were exposed to hypoxia 72h after infection. Apoptotic cells showed positive YO-PRO dye (green), and dead cells showed both positive PI (red) and positive YO-PRO dye (green).

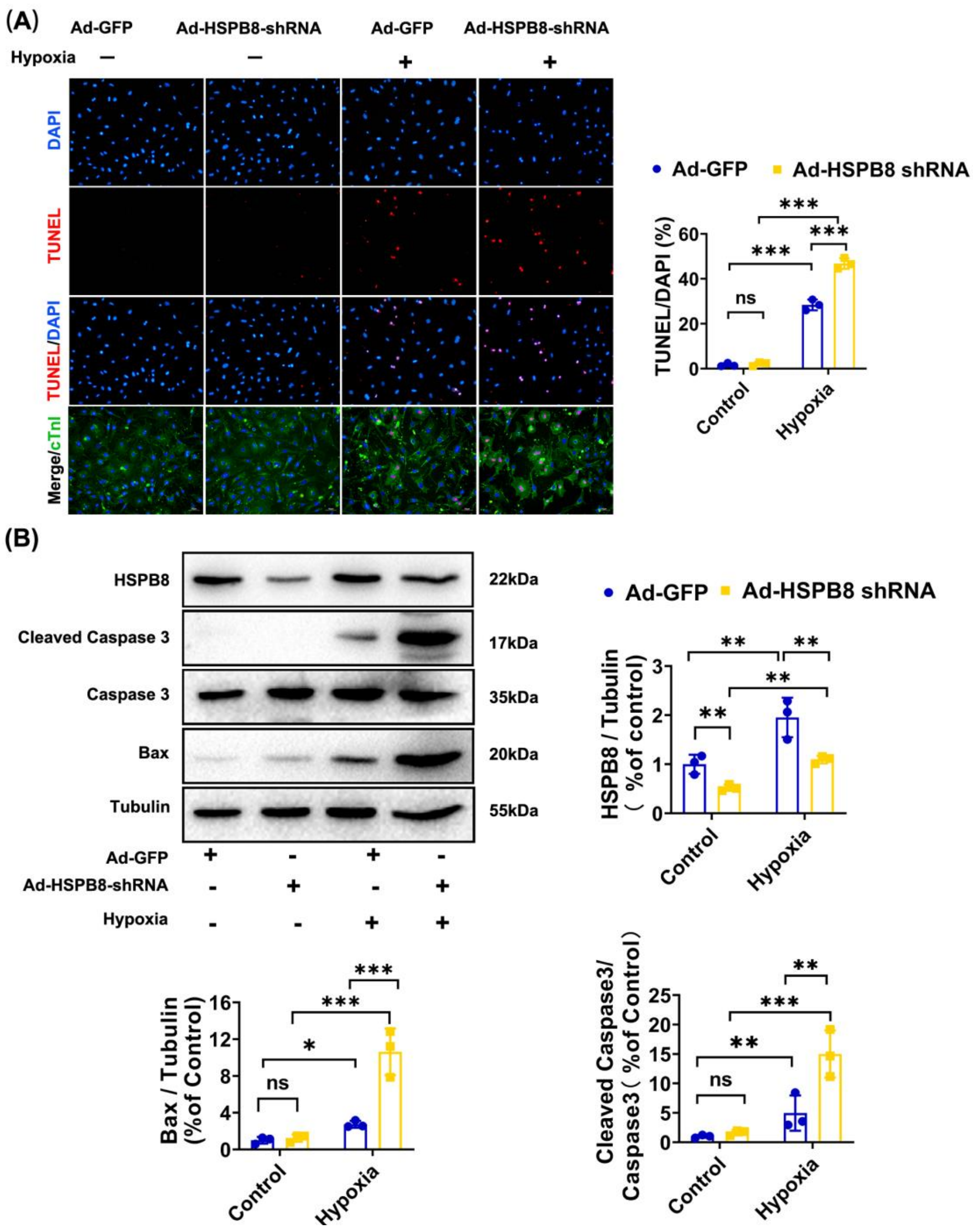

Figure S5. The effect of HSPB8 on apoptosis of cardiomyocytes induced by hypoxia injury. (A)Cardiomyocytes were transfected with Ad-GFP or Ad-HSPB8 shRNA, then were exposed to hypoxia at 72h after infection. TUNEL staining was performed to assess the apoptotic cells at 24h after hypoxia. Representative images of TUNEL were shown in left, and quantification of the apoptosis rate was shown in right (n=3). (B)Knockdown of HSPB8 aggravated the expression of CC3 and Bax induced by hypoxia. Cardiomyocytes were treated as described in (A). The expression of CC3 and Bax were analyzed 24h after hypoxia injury (n=3). ns=no significant, ***P<0.001 vs. Ad-GFP control group. CC3: cleaved caspase-3.

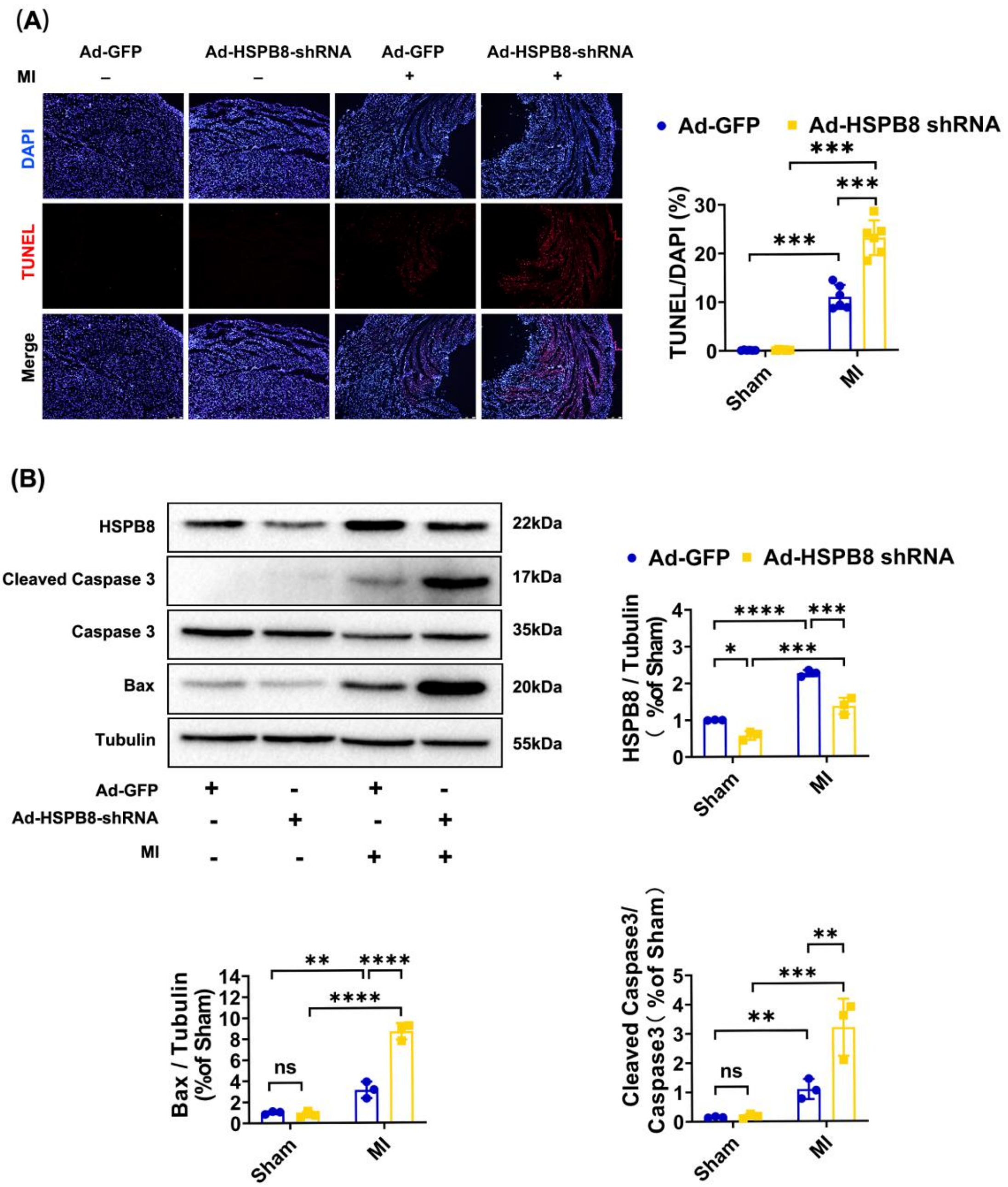

Figure S6. The effect of HSPB8 on myocardial apoptosis induced by AMI. (A)Mice were injected with Ad-GFP or Ad-HSPB8 shRNA, and then were subjected to LAD ligation at 72h after intramyocardial injection. TUNEL staining was performed to assess the apoptotic cells at 24h after hypoxia. Representative images of TUNEL were shown in left, and quantification of the apoptosis rate was shown in right (n=6). (B)Knockdown of HSPB8 aggravated the expression of CC3 and Bax induced by AMI. Mice were treated as described in (A). The expression of CC3 and Bax were analyzed at 24h after MI (n=3). ns=no significant, ****P*<0.001 vs. Ad-GFP sham group. CC3: cleaved caspase-3.

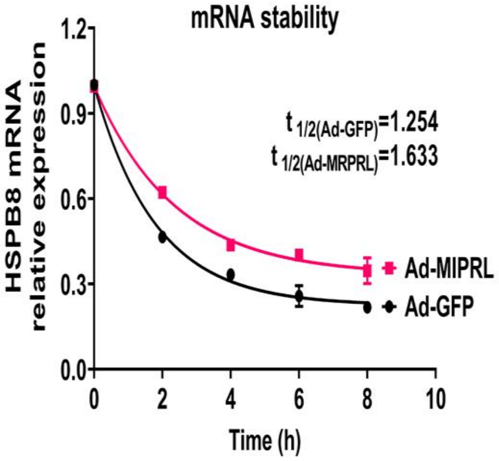

Figure S7. MIPRL increases the stability of HSPB8 mRNA under normoxia. The increase of stability of HSPB8 mRNA by MIPRL was assessed via mRNA stability assay. Cardiomyocytes were transfected with either Ad-GFP or Ad-MIPRL, and subsequently, Actinomycin D (10 μg/mL) was added to the culture medium. The cells were further incubated for 2 to 8 hours. Decay curves of HSPB8 mRNA was generated by Graphpad Prism.

Table 1 The information of primer and probes

**PCR Primers**	
**Homo sapiens**	
MIPRL	CTTGAACTCTCCTGCTCCT
	TCTACTTCTGGCGGTGTG
18S	GTAACCCGTTGAACCCCATT
	CCATCCAATCGGTAGTAGCG
**Mus musculus**	
MIPRL	GCACATGGGTGCTGGAGATT
	AGTGGGAAGGGCAGAAGGGT
HSPB8	GCAGTTAATGCAAGAGTTGCTTTTC
	CCAATGAGTCCCATCAACCAA
18S	CGGCTACCACATCCAAGGAA
	AGCTGGAATTACCGCGGC
**Probe Names**	
MIPRL_FISH	TAGCACAGCCACAGGATAGA
HSBP8 mRNA_FISH	AGAGCGG+TGAGTCCCGAAAG
MIPRL-1_Pull down	CCATGCACTTCTGAGGATTC
MIPRL-2_Pull down	GACCTCCACACGATGCTGTA
MIPRL-3_Pull down	CTCACTTACTCCATTGCGTC
MIPRL-4_Pull down	ATAGTACAACCCATTCTGCC
MIPRL-5_Pull down	TTACAGCTCTGTGTCATCCA
MIPRL-6_Pull down	CCCGCTGACTTTGTTACAAA
MIPRL-7_Pull down	CTATCCTCTGCTTCCAAATC
Scramble_Pull down	TTACCGTCACGATACTGTCG
